# Identification of Compounds With Glucocorticoid Sparing Effects on Suppression of Chemokine and Cytokine Production by Rheumatoid Arthritis Fibroblast-Like Synoviocytes

**DOI:** 10.3389/fphar.2020.607713

**Published:** 2020-12-17

**Authors:** Tadashi Hosoya, Nikunj M. Shukla, Yuya Fujita, Shiyin Yao, Fitzgerald S. Lao, Hiroyuki Baba, Shinsuke Yasuda, Howard B. Cottam, Dennis A. Carson, Tomoko Hayashi, Maripat Corr

**Affiliations:** ^1^ Moores Cancer Center, University of California San Diego, La Jolla, CA, United States; ^2^ Department of Rheumatology, Tokyo Medical and Dental University, Tokyo, Japan; ^3^ Department of Medicine, University of California San Diego, La Jolla, CA, United States

**Keywords:** chemokine, fibroblast, glucocorticoid, high throughput screen, rheumatoid arthritis, synoviocyte, chemotype, cytokine

## Abstract

In recent years target based drug discovery has expanded our therapeutic armamentarium in the treatment of inflammatory and autoimmune diseases. Despite these advances and adverse effects, glucocorticoids remain reliable agents that are used in many of these diseases. The anti-inflammatory mechanisms of glucocorticoids include the suppression of transcription factor activity like nuclear factor kappa B (NF-κB). By reanalyzing data from two prior high throughput screens (HTS) that utilized a NF-κB reporter construct in THP-1 cells, we identified 1824 small molecule synthetic compounds that demonstrated NF-κB suppressive activities similar to the glucocorticoids included in the original >134,000 compound libraries. These 1824 compounds were then rescreened for attenuating NF-κB activity at 5 and 16 h after LPS stimuli in the NF-κB THP-1 reporter cells. After a “Top X” selection approach 122 hit compounds were further tested for toxicity and suppression of LPS induced CXCL8 release in THP-1 cells. Excluding cytotoxic compounds, the remaining active compounds were grouped into chemotype families using Tanimoto based clustering. Promising representatives from clustered chemotype groups were commercially purchased for further testing. Amongst these index compounds a lead chemotype: 1*H*-pyrazolo [3,4 *d*] pyrimidin-4-amine, effectively suppressed CXCL8, and TNF production by THP-1 cells when stimulated with LPS, TNF or IL-1ß. Extending these studies to primary cells, these lead compounds also reduced IL-6 and CXCL8 production by TNF stimulated fibroblast-like synoviocytes (FLS) from rheumatoid arthritis (RA) patients. Importantly a lead 1*H*-pyrazolo [3,4 *d*] pyrimidin-4-amine compound demonstrated synergistic effects with dexamethasone when co-administered to TNF stimulated THP-1 cells and RA FLS in suppressing chemokine production. In summary, a cell based HTS approach identified lead compounds that reduced NF-κB activity and chemokine secretion induced by potent immunologic stimuli, and one lead compound that acted synergistically with dexamethasone as an anti-inflammatory agent showing a dose-sparing effect.

## Introduction

The development of immunosuppressive drugs and target-based drugs has expanded our therapeutic armamentarium in the treatment of inflammatory and autoimmune diseases (Mina-Osorio, 2015; Romão and Fonseca, 2019). Despite these advances, glucocorticoids (GCs) remain the most reliable agents as an initial treatment in the acute phase of the disease and the maintenance therapy for preventing disease relapse GCs are a double-edged sword because long-term use can induce adverse events, including cardiovascular disease, osteoporosis, cataracts and muscle atrophy, in addition to the risk of serious infections (Fardet et al., 2015; Hardy et al., 2020). To minimize the adverse events of GCs, the American College of Rheumatology (ACR) and European League Against Rheumatism (EULAR) established guidelines regarding the clinical use of GCs in rheumatic diseases (Duru et al., 2013; Dejaco et al., 2015; Fanouriakis et al., 2019; Hellmich et al., 2020). To reduce side effects several clinical trials examined whether the use of the conventional immunosuppressive drugs could induce remission without oral GC use (Condon et al., 2013; Gracia-Tello et al., 2017). Combined these studies reported that the management of rheumatic diseases still relies on GCs as part of the therapeutic regimen with its relatively non-specific, but strong anti-inflammatory effects. Hence there remains a clinical need for drugs which can dose-spare or replace the anti-inflammatory effects of GCs.

One of the key mechanisms for the anti-inflammatory effect of GCs is the regulation of nuclear factor kappa B (NF-κB) through IKKß. NF-κB is an essential transcription factor induced by inflammatory responses and plays critical roles in cell cycle progression, cell survival, adhesion, and inhibition of apoptosis (Zhang et al., 2017). Several human genetic diseases confirm the multifunction of NF-κB including genetic defects in NF-κB activating molecules (e.g., NEMO) resulting in an immunodeficiency phenotype (Döffinger et al., 2001; Pannicke et al., 2013) and in NF-κB regulatory molecules (e.g., A20/TNFAIP3, OTULIN) which causes an autoinflammatory phenotype (Damgaard et al., 2016; Zhou et al., 2016). When NF-κB is activated, its activation is transient and regulated by the consumption of downstream adaptor molecules and the induction of anti-inflammatory molecules. In chronic inflammatory diseases, such as rheumatic diseases, auto-inflammatory diseases, and inflammatory bowel diseases, excessive and continuous activation of NF-κB are common findings reflecting a large amount of inflammatory stimuli and the dysregulation of negative-feedback mechanisms (Brown et al., 2008; Liu et al., 2017).

GC’s anti-inflammatory mechanisms were attributed to inhibitory effects against NF-κB by interfering with DNA binding competitively and inducing anti-inflammatory genes (Xavier et al., 2016; Hardy et al., 2020). Also, several immunosuppressants and disease-modifying anti-rheumatic drugs (DMARDs), such as calcineurin inhibitors (Du et al., 2009), iguratimod (Aikawa et al., 2002), and methotrexate (Spurlock et al., 2015)**,** attenuated NF-κB activity at least indirectly. These findings indicate that at least partial inhibition of NF-κB signaling pathway remains a promising therapeutic strategy. However, despite the intensive effort to discover and develop NF-κB targeting drugs, few agents have been approved for clinical use because of unexpected adverse events, including nephrotoxicity, neuropathy, and paradoxical IL-1ß release (Gilmore and Herscovitch, 2006; Greten et al., 2007; Mina et al., 2016).

In this study, we identified novel compounds that suppressed NF-κB signaling using a fluorescence resonance energy transfer (FRET) based reporter in the human monocytic leukemic cell line, THP-1. We used the data from two prior high throughput screens (HTS) that used this same reporter line and a largely overlapping chemical library (Chan et al., 2013; Chan et al., 2017). The first screen (HTS1) examined the direct effect of the compounds on the NF-κB FRET activity after 5 h of treatment and the second screen (HTS2) examined the effect of the compounds on LPS induced NF-κB FRET activity after 12 h of treatment. Hit compounds were selected on the basis of the ability to reduce NF-κB signaling and to sustain a reduction in NF-κB signaling after a primary inflammatory stimulus, lipopolysaccharide (LPS). After the confirmation screens, we identified chemotype clusters that suppressed LPS-induced NF-κB activation and subsequently selected hit candidates which were enriched in the larger chemotype families and demonstrated minimal toxicity. Selected compounds were tested for biological effects of reducing cytokine and chemokine production resultant from a primary inflammatory stimulus to the monocytic cell line, THP-1, and fibroblast-like synoviocytes (FLS) from rheumatoid arthritis (RA) patients and synergy with dexamethasone.

## Methods

### Cell Lines and Reagents

The CellSensor^®^ NF-κB-bla human monocytic THP-1 cell line was purchased from Thermo Fisher Scientific (Waltham, MA). THP-1 cells were purchased from American Type Culture Collection (ATCC, Manassas, VA). Cells were cultured in RPMI medium (Thermo Fisher Scientific, Waltham, MA) supplemented with 10% FBS (Omega Scientific Inc., Tarzana, CA), 100 U/mL penicillin, 100 μg/mL streptomycin (Thermo Fisher Scientific, Waltham, MA), and 55 μM ß-mercaptoethanol (SigmaAldrich, St. Louis, MO).

Rheumatoid arthritis fibroblast-like synoviocytes (RA FLS) were isolated from synovial tissues derived from patients with RA when they underwent joint replacement surgery or synovectomy as described previously (Nonomura et al., 2006). Patients were age ≥18 years with active RA based on the ACR 1987 Revised Criteria (Arnett et al., 1988) and consent forms were completed by the patients before surgery. The study protocol was approved by the institutional review board at Tokyo Medical and Dental University, Tokyo, Japan and are in accordance with the principles of the Declaration of Helsinki. RA FLS were cultured in DMEM supplemented with 10% FBS, 100 U/mL penicillin, and 100 µg/mL streptomycin in a humidified 5% CO_2_ incubator. All experiments used proliferating RA FLS (from passages 5–11).

LPS (*Escherichia coli* 0111:B4, Sigma-Aldrich, St Louis. MO) was used in the HTS, and LPS-EB Ultrapure (InvivoGen, San Diego, CA) was used in the confirmation screens and subsequent studies. Human TNF (Thermo Fisher Scientific, Waltham, MA and R&D systems, Minneapolis, MN), IL-1ß (Promega, Southampton, United Kingdom), dexamethasone (DEX, Fresenius Kabi Usa, Lake Zurich, IL and MP Biomedicals, Solon, OH) and 5-(4-fluorophenyl)-2-ureidothiophene-3 carboxylic acid amide (UTC; Toronto Research Chemicals, Inc., Ontario, Canada), a known IKK inhibitor (Endo et al., 2007) were commercially purchased.

Hit compounds were purchased from ChemBridge (San Diego, CA) and ChemDiv (San Diego, CA) and dissolved in dimethyl sulfoxide (DMSO, Sigma Aldrich, St Louis. MO; Supplemental Table S1). Purity of the compounds was verified as >95% by LC-MS. Endotoxin levels were less than 10 EU/μmol by EndoSafe^®^ (Charles River Laboratory, Wilmington, MA).

### NF-κB Activation Assay Using Reporter Cells

The CellSensor^®^ NF-κB-bla THP-1 cell line has a stably integrated ß-lactamase reporter gene under the control of the nuclear factor kappa B (NF-κB) response element. LPS induced NF-κB activation resulted in ß-lactamase production. In the absence of ß-lactamase activity, excitation of the coumarin at 409 nm in the ß-lactamase substrate (LiveBLAzerTM-FRET B/G (CCF4-AM), Thermo Fisher Scientific) resulted in emission at 520 nm. In the presence of ß-lactamase, CCF4 was enzymatically cleaved and excitation at 409 nm produced a blue fluorescence signal (at 450 nm). The CellSensor^®^ NF-κB-bla THP-1 cells were dispersed in 96-well plates (5 × 10^4^ cells/200 µL/well) and incubated for 4 h. Then the cells were treated with 5 µM of each compound and 10 ng/ml of LPS for 5 or 12 h in 5% CO_2_ at 37°C. After incubation, the ß-lactamase substrate mixture (prepared according to the manufacturer’s protocol) was added to each well. Plates were incubated at room temperature in the dark for 2 h. Fluorescence was measured on a Tecan Infinite M200 plate reader (Tecan, Mannedorf, Switzerland) at an excitation wavelength of 405 nm and emission wavelengths of 465 and 535 nm. Emission ratios are calculated by dividing values from emission wavelength of 465 nm by those from emission wavelength of 535 nm. The response ratio was calculated as follows [(emission ratio of a test well)/average emission ratio of wells with vehicle (0.5% DMSO)] and values were normalized to the LPS control treated wells [response ratio of the compound/response ratio of LPS].

### Cell Viability Assays

Two types of tetrazolium were used for viability assays. For THP-1 cells, the cells were dispensed in 96-well plates (10^5^ cells/200 µL/well) and treated with 5 µM of each compound. After 18 h of incubation, 0.5 mg/ml 3-[4,5-dimethylthiazol-2-yl]-2,5-dipheyl tetrazolium bromide (MTT; Thermo Fisher Scientific) in assay media was added to each well at a final concentration of (0.5 mg/ml) and further incubated for 4–6 h. The absorbances at 570 and 650 nm were measured by a Tecan Infinite M200 plate reader. For RA FLS, the cells were dispersed into 96-well flat bottom plates (10^4^/200 µL/well) and incubated overnight. The next day, RA FLS were treated with 5 µM of each compound for 18 h of incubation. Ten µl of WST-8 [2-(2-methoxy-4-nitrophenyl)-3-(4-nitrophenyl)-5-(2,4-disulfophewnyl)-2H-tetrazolium, monosodium salt] solution was added to each well and the cells were further incubated for 2–4 h. The absorbance was measured at 450 nm with Bio-Rad iMark plate reader (Bio-Rad Laboratories Inc., Hercules, CA, Unites States).

For apoptosis studies, THP-1 cells (10^5^ cells/200 µL/well) were plated in 96 well plates and pre-treated with 5 µM compound, 5 µM DEX, or 5 μM UTC; or vehicle (DMSO) for 1 h and then LPS (10 ng/ml) was added. After 24 h incubation the cells were washed twice with cold PBS and then resuspended in Annexin V Binding Buffer (BD Pharmingen, Mountain View, CA) and 5 × 10^4^ cells in 50 µL were transferred to a V bottom plate. The cells were stained with FITC-Annexin V (BD Pharmingen) and 7-Amino-Actinomycin (7-AAD; BD Pharmingen) for 15 min and evaluated by flow cytometry (MACSQuant^®^ Analyzer 10, Miltenyi Biotec, Germany). Data were analyzed using FlowJo software (FlowJo LLC, Ashland, OR).

### Cytokine and Chemokine Production Assays

THP-1 cells (10^5^ cells/200 µL/well) and RA FLS (10^4^/200 µL/well) were plated in 96 well plates and pre-treated with compound or vehicle for 1 h and then stimulated with either LPS (10 ng/ml), IL-1ß (2 ng/ml) or TNF (2 ng/ml for THP-1 and 1 ng/ml for RA FLS). After 18 h of incubation, supernatants were collected and the levels of cytokines and chemokines in the culture supernatants were measured by ELISA according to the manufacturer’s protocols (R&D systems, Minneapolis, MN).

### Compound Clustering Into Chemotypes

The structures of the compounds (simplified molecular-input line-entry system format, SMILES) were subjected to substructure-based clustering using the server based ChemMine tools (University of California, Irvine; http://chemmine.ucr.edu/tools/launch_job/Clustering/) and binning clustering application with a similarity cutoff of 0.5.

### Drug Synergy Analysis

Drug synergy analysis was performed using Isobologram plots which were calculated according to reported procedures using methods of Chou-Talalay (Chou and Talalay, 1984) to compute IC_50_ and determine synergism. IC_50_ is computed from the median effect equation. Synergism analysis is carried out using the Combination index (CI)-isobol method. Data analysis was performed using the CompuSyn software available on combisyn.com. Detailed methodology was used as described in a prior report (Chou, 2006). Briefly, the median-effect equation is computed to obtain the linear regression for the effect of inhibitors DEX and 1–1 as F_a_/F_u_ = (D/D_50_)^m, where *D* is the dose, *F*_*a*_ and *F*_*u*_ is the fraction of the inhibition and uninhibited response by the dose D (F_a_ + F_u_ = 1); D_50_ is the dose producing the median effect (i. e, IC_50_). The constant *m* determines the shape of the dose-effect curve. The median-effect equation in logarithmic form is log (F_a_/F_u_) = m log(D)–m log (D_50_) which essentially represents a linear relationship between log(F_a_/F_u_) and log(D). Thus, linear regression curves are obtained with the observed inhibition data for the individual inhibitors to obtain estimated values for the parameters *m* and D_*50*_. This is followed by CI-isobol method to quantitatively assess the synergism between these inhibitor drugs. A combination index (CI) is estimated from dose-effect data of single and combined drug treatments. A value of CI less than one indicates synergism; CI = 1 indicates additive effect; and CI > 1 indicates antagonism. Drug interaction (synergism or antagonism) is more pronounced the further a CI value is from 1. Formally, the combination index (CI) of a combined drug treatment is defined as CI = D_1_/Dx_1_ + D_2_/Dx_2_. Here D_1_ and D_2_ are the doses of DEX and 1-1*,* respectively, in the combination; Dx_1_ and Dx_2_ each is the dose of a treatment with only DEX and 1-1 that would give the same effect as that of the combination, respectively. The doses Dx_1_ and Dx_2_ were estimated from the median effect equation above for single drug treatments. From the median effect equation, the estimated dose (i.e., D) necessary to produce the inhibition (i.e. F_a_, F_u_) obtained by the combination was calculated. The results are presented as a normalized isobologram. A point in the isobologram represents the effect of a drug(s) treatment. The further a point lies from the additive line, the larger the difference between one and its CI, hence the stronger is the synergistic effect.

## Results

### Overall Screening Strategy and Design

**FIGURE 1:**
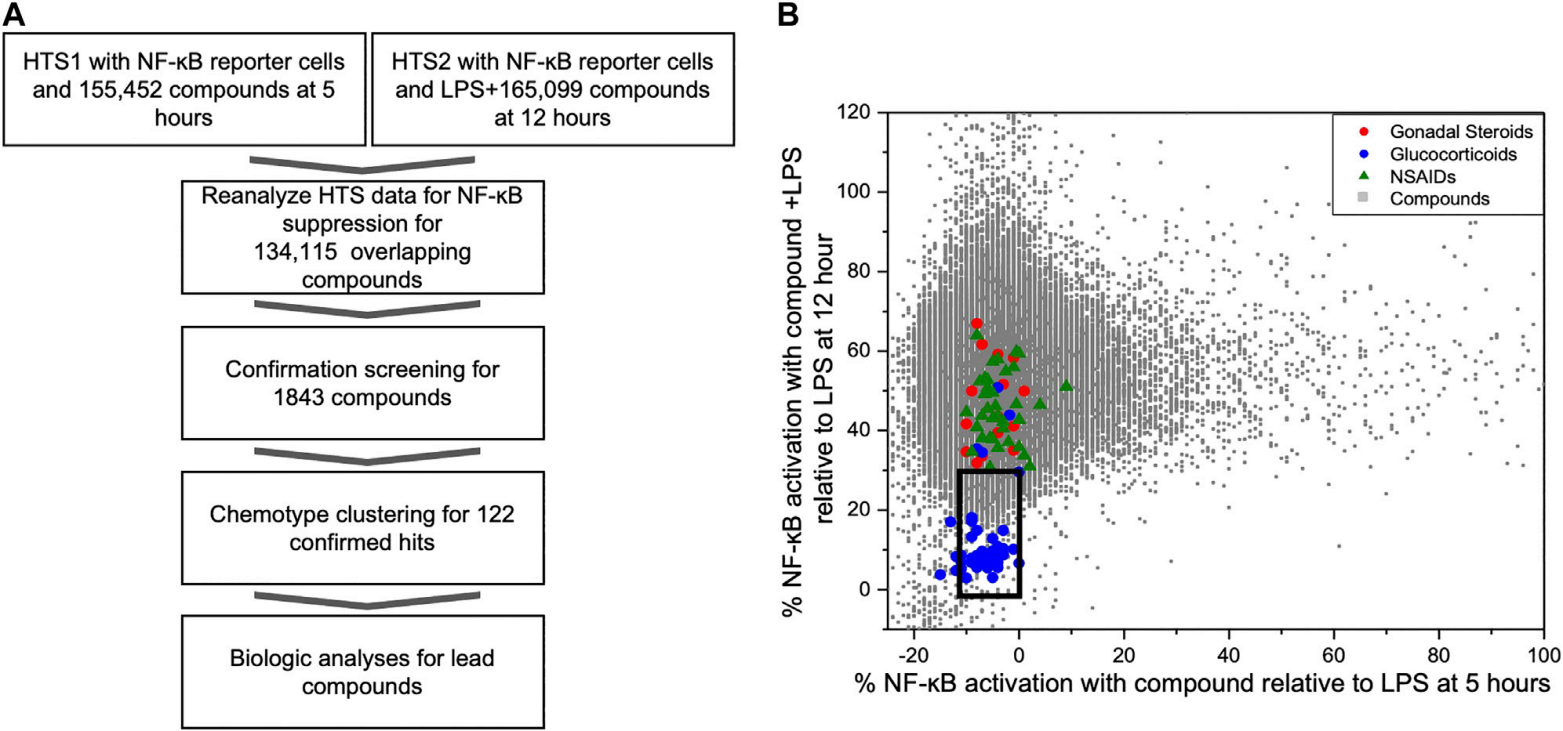
Identification of NF-κB suppressive compounds; **(A)** Workflow strategy for identifying NF-κB suppressive compounds by mining existing HTS data and subsequent screening strategy; **(B)** Distribution of NF-ĸB reporter activity from compounds screened in two prior HTS relative to known bioactive compounds. We conducted two prior HTS using the same cell-based FRET assay with THP-1 CellSensor NF-κB reporter cells. The first tested the activation/inhibition of reporter signal with compound treatment at 5 h (HTS1, *x*-axis), and other tested the reporter signal with LPS plus compound at 12 h (HTS2, *y*-axis), using 155,452, and 165,099 compound libraries, respectively. Results of the 134,115 overlapping compounds were normalized to the LPS controls in each assay and are plotted individually. Compounds in an area (black box) encapsulating known GC and below the activity of gonadal steroids or COX inhibitors (*X* axis -10%–0% and *Y* axis <30%) were picked for further assessment of immunosuppressant activity.

As part of our compound identification strategy (Figure 1A) we re-analyzed data from two prior HTS that we had previously conducted using CellSensor NF-κB-bla THP-1 reporter cells and compound libraries that were acquired at two different times (5 years apart) from the University of California, San Francisco, Small Molecule Discovery Center (SMDC: https://smdc.ucsf.edu) (Chan et al., 2013; Chan et al., 2017). We determined an area that bounded the activities of the named GCs in overlapping subset of the libraries and identified 1824 compounds that attenuated NF-κB activities in both HTS within the perimeter of this area (box in Figure 1B). We then performed a series of confirmation screens with these hit compounds (5 µM) for their effects on the kinetics of NF-κB activity in LPS stimulated reporter cells at peak (5 h) and decay (16 h) timepoints. There were 122 compounds that met the following criteria: NF-κB activity <50% max at 5 h or <25% max at 16 h. These compounds were then evaluated for effect on IL-8 production and cellular toxicity by MTT assay in THP-1 cells. Excluding compounds with <90% viability by MTT assay, the remaining compounds were clustered into chemotype families. Candidate compounds were purchased from commercial vendors to represent chemotype families with the largest number of active members and to represent chemical diversity. After purchasing candidate compounds, we analyzed further biological activities using THP-1 cells and synovial fibroblasts from rheumatoid arthritis patients (RA FLS) for primary activity and potential synergism with dexamethasone.

### Re-analysis of Existing High Throughput Screening Data

In prior studies, two HTS were conducted to identify novel compounds that initiated or sustained innate immune activation via the NF-κB pathway using CellSensor NF-κB-bla reporter containing THP-1 cells (Chan et al., 2013; Chan et al., 2017). The libraries for these studies came from the SMDC and had 134,115 overlapping compounds and LPS was used as a control on each plate in both studies. The reporter cells were incubated with compound alone (5 µM) for 5 h in the first HTS (HTS1). In the second HTS (HTS2), the reporter cells were incubated with compound (5 µM) in the presence of LPS (100 ng/ml) for 12 h as a primary stimulus. The FRET activity was normalized to the LPS controls on each plate in the respective HTS. The normalized activities of individual compounds were plotted for the activities after 5 h (compound alone in HTS1, *x*-axis) or 12 h (compound + LPS in HTS2, *y*-axis) incubation (Figure 1B). The chemical collection from the SMDC contained compounds with known drug properties including several GCs, gonadal steroids and cyclo-oxygenase (COX) inhibitors. To segregate compounds with the most potential as immunosuppressants, we chose the compounds with similar activity to the glucocorticoid cluster region and were excluded from the region populated with non-steroidal anti-inflammatory drugs (NSAIDS) or gonadal steroids for further screening (Figure 1B). A total of 1824 compounds were selected from the glucocorticoid region of the combined HTS data that fit the activation thresholds set at each respective time point.

### Confirmation Screen and Kinetic Profiling of NF-κB Activity

**FIGURE 2:**
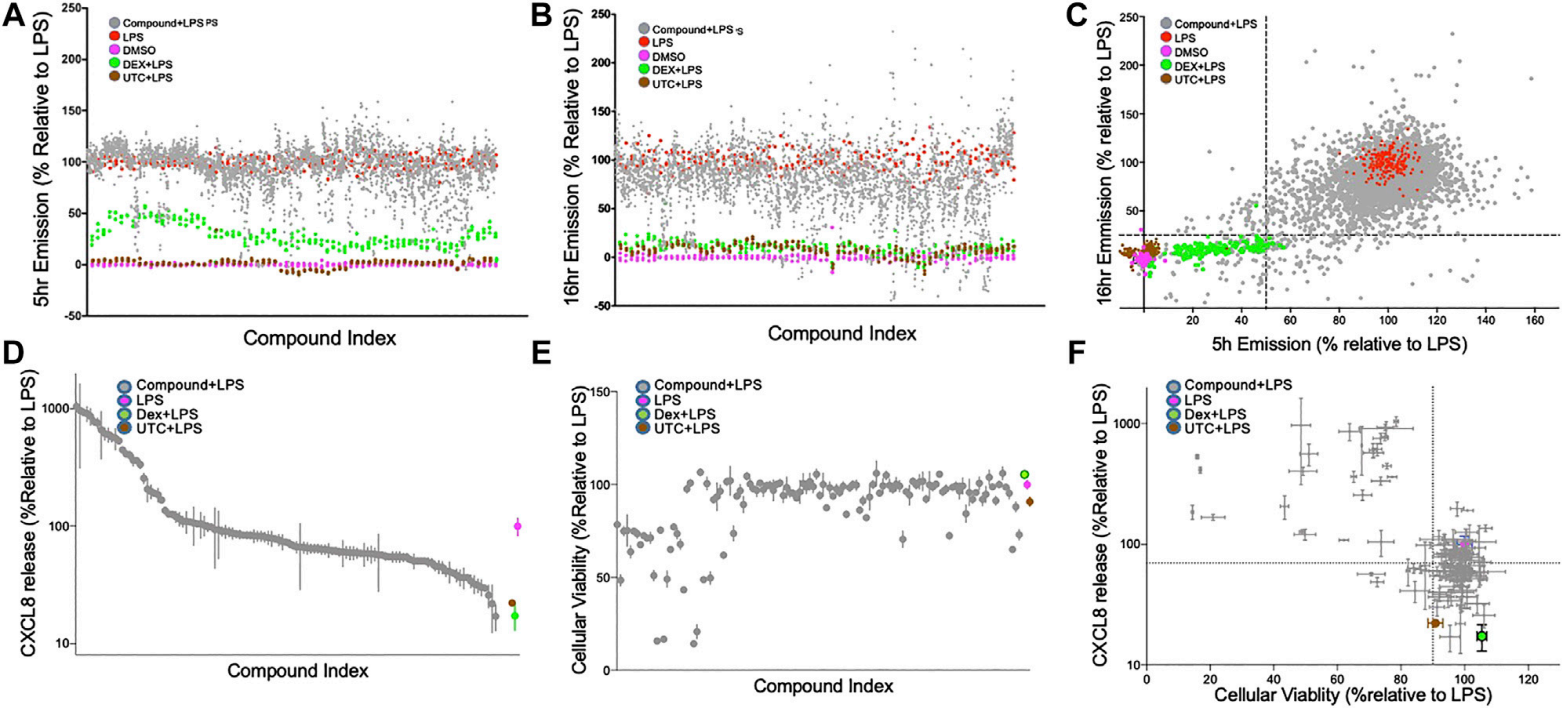
Confirmation screen of compounds for NF-κB reporter activity, viability and chemokine stimulation; **(A–C)**: Confirmation screening of initial 1824 hit compounds. Inhibition of an LPS induced signal by compound as a percent NF-ĸB activity relative to LPS controls at 5 h; **(A)**, 16 h; **(B)** and both; **(C)**. 1824 compounds were assessed with LPS as the primary stimulus using the FRET assay with THP-1 CellSensor NF-κB reporter cells. The percent NF-κB activation was calculated relative to DMSO + LPS (red) as 100% and DMSO as vehicle control (magenta) as 0%. All assay runs included DEX + LPS (green), and UTC + LPS (brown) as positive controls. Gray dots represent the duplicate data of each of the tested compounds. 122 compounds were selected based on the “Top-X″ criteria: < 25% at 16 h or <50% at 5 h (dotted lines) **(D and E)**. Relative CXCL8 production and cellular viability of THP-1 cells treated with the 122 selected compounds. THP-1 cells treated with LPS (10 ng/ml) and 122 hit compounds (5 µM) were analyzed for CXCL8 production by ELISA; **(D)**, and tested for cellular toxicity by MTT assay; **(E)**. Results were normalized relative to the LPS controls, and ranked by CXCL8 production. The compounds in **(D)** and **(E)** are presented in the same order. **(F)** Both assays are plotted and the dotted line indicates 70% production of CXCL8 and 90% viability relative to the control. DEX (blue) and UTC (brown) were used as positive controls. Data shown as mean ± SD of triplicate data.

The 1824 compounds that were identified by the data mining strategy to reduce NF-κB signaling were then rescreened in duplicate using the same THP-1 CellSensor NF-κB-bla reporter cells. In prior work we established conditions where LPS (10 ng/ml)-induced NF-κB activity which peaked at 5 h and decayed to 60% at 16 h after stimulation. Using LPS as a primary inflammatory stimulus the effect of these compounds on NF-κB activity was evaluated at two time points to profile the kinetics of their suppression (Chan et al., 2017). Analysis of the compound behavior at the 5 h time point against the 16 h time point shows a higher number of compounds with lower NF-κB activity following a longer incubation time vs. the shorter time (Figure 2A,B). These confirmation screens included dexamethasone (DEX), and 5-(4-fluorophenyl)-2-ureido-thiophene-3 carboxylic acid amide (UTC) as controls that suppress NF-κB activity with distinct mechanisms. As expected, DEX inhibited NF-κB activity more potently at 16 h than 5 h, and UTC inhibited NF-κB activity at both time points. To select possible immunosuppressants as hit compounds we utilized a naïve standard activity-based approach by selecting compounds with a defined activity threshold (frequently called a “Top X″ approach). We first excluded the known bioactive compounds and then set the desired activity threshold levels of NF-κB activity at ≤ 50% or ≤25% of the normalized FRET emission ratios at 5 and 16 h, respectively (Figure 2C). This area encapsulated most of the DEX controls and 122 unique compounds which were selected for further assessment (Figure 2D–F).

### Evaluation of Cytotoxicity and Suppression of Chemokine Production in THP-1 Cells

The 122 hit compounds underwent additional screening and were evaluated for their effects on cell viability and the ability to suppress production of an NF-κB-associated chemokine, CXCL8 (IL-8) by LPS stimulated THP-1 cells (Figure 2D). The cells were also examined for viability after 24 h of stimulation by MTT assay (Figure 2E). Relative CXCL8 production and cellular viability by the treatment candidate compounds, DEX, and UTC were normalized to the LPS + vehicle controls (Figure 2D–F). Compounds that showed low cytotoxicity (>90% viability) and suppressed CXCL8 production at 70% or lower relative to the control were brought forward as potential candidates for future evaluation as immunosuppressants (Figure 2F). The 122 hit compounds were also tested for apoptosis induction in the presence of LPS (Supplemental Figure S1). The % live cells in the apoptosis assay and the % viable cells in the MTT assay correlated with a Pearson r coefficient of 0.85 (*p* < 0.0001). However there were six compounds that demonstrated >90% viability in the MTT assay, but had >10% apoptotic cells in the apoptosis assay indicating that the two assays provided complementary assessments.

### Chemotype Clustering and Validation of Lead Compounds

Of the 122 hit compounds 74 were clustered into 18 chemotypes based on their molecular similarities and common scaffolds using Tanimoto indexes and 48 compounds were singletons (compounds not associated with a chemotype; Supplemental Figure S2). The 51 compounds that met all of the biological selection criteria (90% viability and 70% CXCL8 release) consisted of 11 chemotypes and included 17 singletons (Table 1). Three of the eleven chemotypes were excluded based on the potential for them to be Michael acceptors. In many cases α,β-unsaturated carbonyls such as those found in these chemotypes can form adducts with thiols, especially under physiological conditions (pH = 6–8), reducing the *in vivo* efficacy (Jackson et al., 2017). Also, Michael acceptors are often reversible IKKß inhibitors and thereby inhibit NF-κB response (Rossi et al., 2000; Karin et al., 2004). Thus, from the remaining chemical families, ten compounds from 5 chemotypes were purchased to validate and further assess as they had multiple hits within the chemotype cluster and represented chemical diversity between the scaffolds. These compounds were reassessed for their suppression of CXCL8 production and cytotoxicity in THP-1 cells (Table 2). Six inhibitors from three chemotypes reduced the level of CXCL8 levels to 70% or less than that of the LPS control. However, the other four compounds from chemotypes 13 and 17 did not meet the set criteria and were inconsistent with the data previously seen from the compounds obtained from the original HTS library. These discrepancies could be due to the age of the DMSO stocks in the HTS samples permitting degradation, precipitation or other unknown modifications. LC-MS analysis of the purchased compounds showed that the purity of the material (>95% by HPLC) was sufficient to validate the observed activity.

**TABLE 1:** Number of compounds in lead chemotype clusters by screening stage.

Chemotype cluster number	Chemotype	Number in 1824 starting compounds	Number passed all selection criteria	Selection or exclusion criteria
1	1*H*-pyrazolo [3,4 *d*] pyrimidin-4-amine	22	11	Selected
2	(*E*)-5-benzylidene imidazolidine-2,4-dione	17	4	Excluded as a michael acceptors
3	Bis-aryl urea	12	1	Selected
5	2-Nitro furan arylamide	9	5	Selected
6	Piperidine derivatives	9	2	Weak inhibition
8	(*E*)-3-phenyl-1-(quinolin-3-yl)prop-2-en-1-one	8	3	Excluded as a michael acceptor
10	1*H*-pyrazolo [3,4-*b*] quinolin-3-amine	6	2	None commercially available
13	2-Imino-1,2-dihydro-5*H*-dipyrido [1,2-*a*:2′,3′-*d*]pyrimidin-5-one	5	3	Selected
15	Piperazine derivatives	5	1	None commercially available
16	*(E*)-3-phenyl-1-(pyridin-3-yl)prop-2-en-1-one	3	1	Excluded as a michael acceptors
17	Thiophene pyrazine piperidine analogs	3	2	Selected
18	Pyrazole derivatives	22	2	None commercially available
n/a	Singleton compounds	n/a	14	Excluded

**TABLE 2:** Validation of purchased candidate compounds.

Compound number	Chemotype	MW (g/mol)	CXCL8 production. Relative to LPS^b^ (original library)	CXCL8 production. Relative to LPS^c^ (purchased)	% Cellular Viability (Purchased)
1–1^a^	1*H*-pyrazolo [3,4 *d*] pyrimidin-4-amine	321	50.6%	47.1%	98.7
1–2^a^	1*H*-pyrazolo [3,4 *d*] pyrimidin-4-amine	295	62.7%	70.1%	100.1
3–1^a^	Bis-aryl urea	261	50.6%	60.7%	105.6
5–1^a^	2-Nitro furan arylamide	276	21.9%	18.8%	98.7
5–2^a^	2-Nitro furan arylamide	300	54.3%	29.8%	101.8
5–3^a^	2-Nitro furan arylamide	312	66.7%	37.7%	100.1
13–1	2-Imino-1,2-dihydro-5*H*-dipyrido [1,2-*a*:2′,3′-*d*]pyrimidin-5-one	454	25.8%	95%	106.1
13–2	2-Imino-1,2-dihydro-5*H*-dipyrido [1,2-*a*:2′,3′-*d*]pyrimidin-5-one	454	32.3%	124.8%	104
13–3	2-Imino-1,2-dihydro-5*H*-dipyrido [1,2-*a*:2′,3′-*d*]pyrimidin-5-one	454	54.8%	129%	101.4
17–1	Thiophene pyrazine peperidine analog	417	30.1%	82.2%	91.3

a Candidate compounds validated in rescreening.

b The mean CXCL8 induced by LPS was 185.3 pg/ml and was normalized as 100%.

c The mean CXCL8 induced by LPS was 201.1 pg/ml and was normalized as 100%.

### Dose-Response of Lead Compounds on the Cytokine and Chemokine Production in Stimulated THP-1 Cells

**FIGURE 3:**
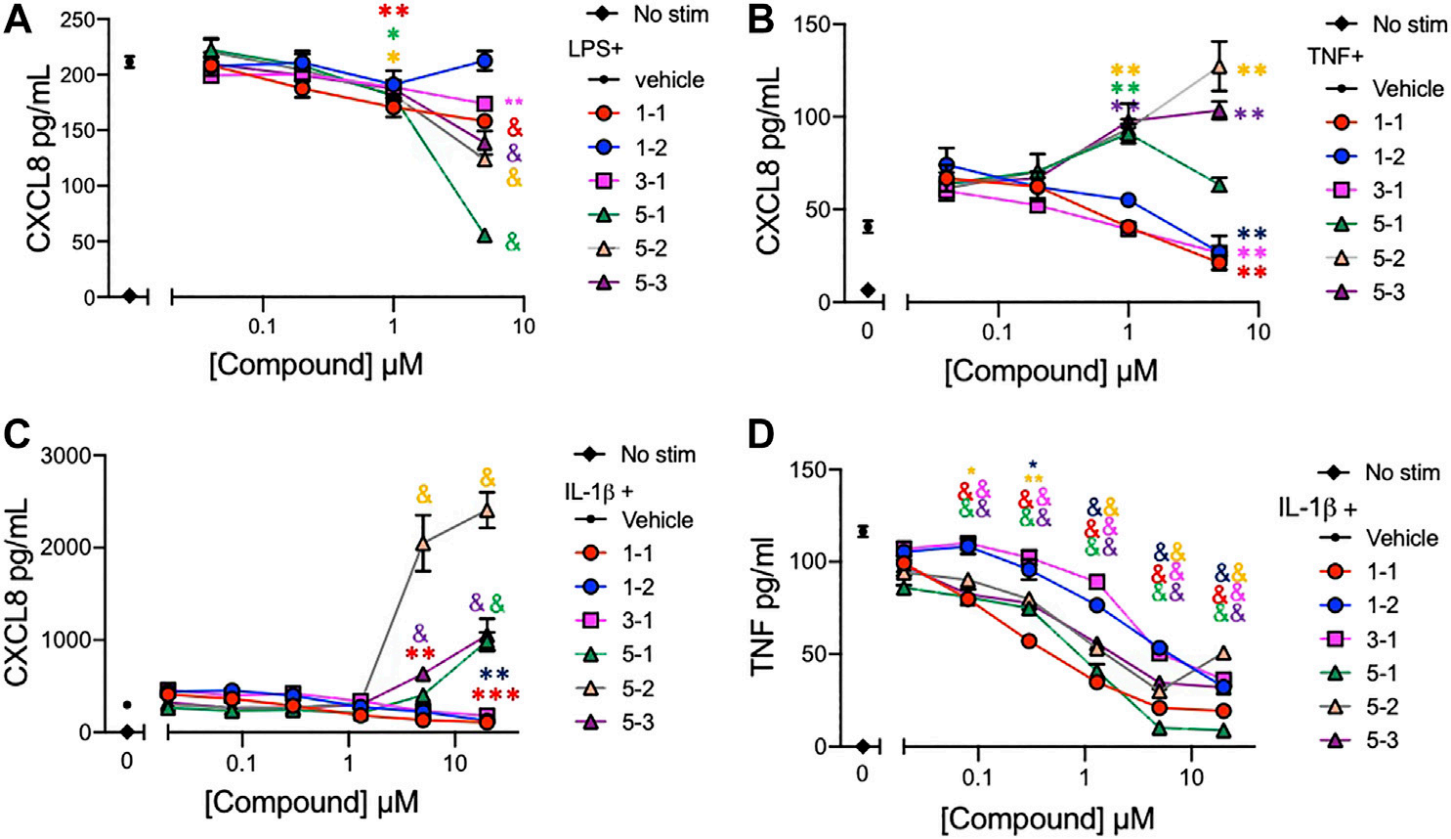
Cytokine and chemokine suppression by the lead compounds in THP-1 cells **(A–C)** CXCL8 production by THP-1 cells treated with graded concentrations of the indicated compounds and stimulated with 10 ng/ml of LPS; **(A)**, 2 ng/ml of TNF; **(B)**, or 2 ng/ml of IL-1ß; **(C)** overnight; **(D)** TNF production from THP-1 cells stimulated with 2 ng/ml of IL-1ß and compounds overnight. Candidate compounds were added at indicated concentrations with 0.1% final concentration of DMSO as vehicle. Shown are mean ± SEM and * indicates *p* < 0.05, **p < 0.01, ***p < 0.001, & *p* < 0.0001 by ANOVA with Dunnett’s post hoc test comparing compound against vehicle + LPS, TNF or IL-1ß respectively. Data are representative of two independent experiments showing similar results.

The lead compounds belonging to chemotypes 1, 3, and 5 were tested for potency in suppressing chemokine and cytokine production by THP-1 cells in the presence of different inflammatory stimuli. The production of CXCL8 induced by either LPS (10 ng/ml), IL-1ß (2 ng/ml), or TNF (2 ng/ml) and TNF induced by IL-1ß (2 ng/ml) was assessed using serially diluted compounds. The compounds except 1–2 reduced the level of CXCL8 production stimulated by LPS in a dose dependent manner (Figure 3A). However, the compounds from chemotype 5 enhanced CXCL8 release by THP-1 cells when stimulated with IL-1ß, or TNF (Figure 3B, C). The IC_50_ for 1–1, 1-2 and 3-1 for TNF stimulated CXCL8 release included 900 nM, 4,130 and 960 nM respectively (Figure 3B). The IC_50_ for 1–1, 1-2 and 3-1 for IL-1ß stimulated CXCL8 release included 400, 1770, and 2020 nM, respectively, (Figure 3C). Interestingly, all of the compounds including those from chemotype 5 reduced TNF release by THP-1 cells when stimulated with IL-1ß (Figure 3D). The IC_50_ for 1–1, 1–2, and 3–1 for IL-1ß stimulated TNF release included 190, 2,770, and 2,420 nM. As an inflammatory tissue environment can have a variety of perpetuating stimuli we opted not to move forward with the compounds from chemotype 5 as they may increase inflammation under certain circumstances.

### The Interaction of Lead Compounds and Dexamethasone

**FIGURE 4:**
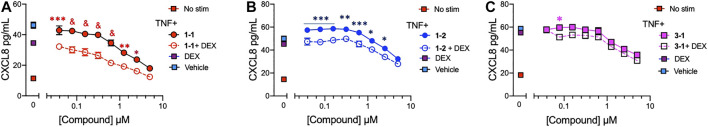
Potency of lead compounds combined with DEX in inhibiting CXCL8 production. Compounds 1–1; **(A)**, 1–2; **(B)** and 3–1; **(C)** were added at the indicated concentrations and DEX was added at 100 nM with 0.04% final concentration of DMSO as vehicle to TNF 2 ng/ml stimulated THP-1 cells overnight. CXCL8 was measured in the supernatant. Data are represented as mean ± SEM and indicates *p* < 0.05, **p < 0.01, ***p < 0.001, & *p* < 0.0001 by two way ANOVA with Bonferroni post hoc test comparing compound vs. compound + DEX. Data are representative of two independent experiments showing similar results.

**FIGURE 5:**
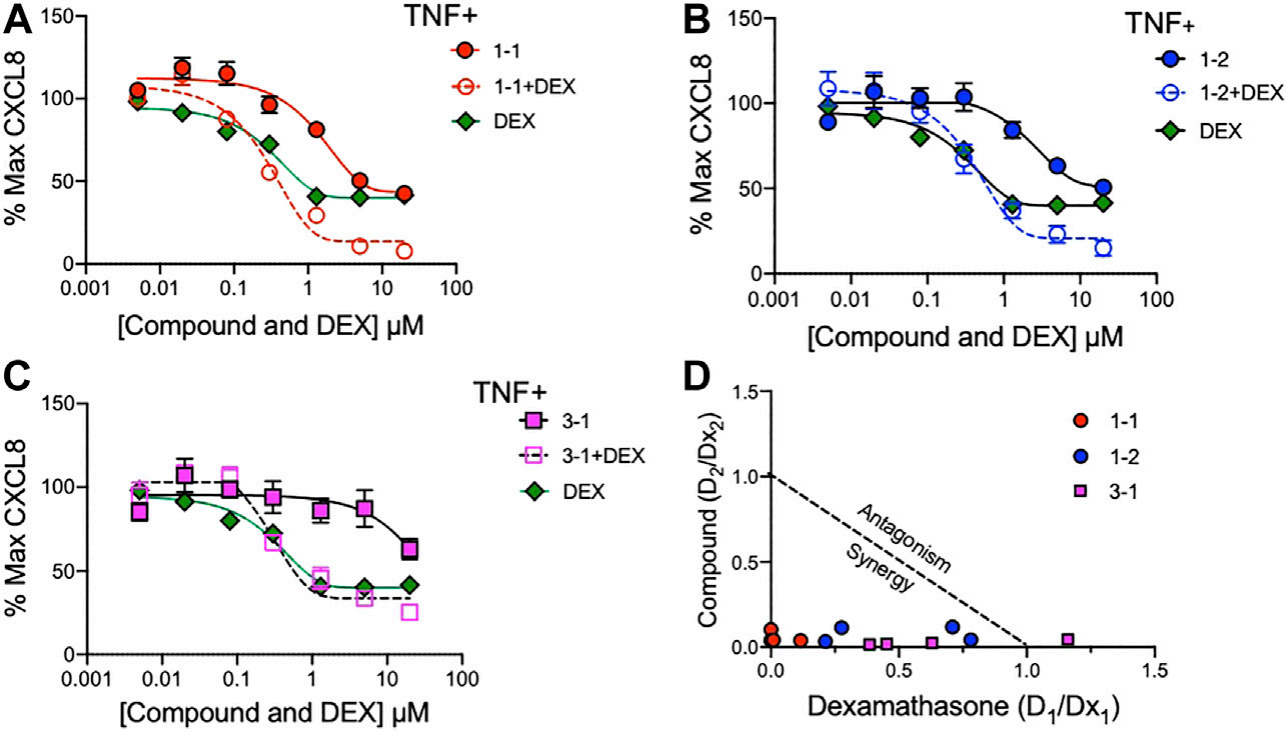
Synergistic effects of lead compounds with DEX in suppressing TNF stimulated CXCL8 production. Compounds 1–1; **(A)**, 1–2; **(B)** and 3–1; **(C)** and DEX were titrated to the indicated concentrations and added to TNF 2 ng/ml stimulated THP-1 cells overnight and CXCL8 measured in the supernatant. Vehicle was 0.04% DMSO. Data are represented as mean ± SEM. **(D)** Potency ratios were calculated and presented as isobolograms. The dotted line represents additivity between DEX and the compounds. Data are representative of two independent experiments showing similar results.

To assess whether the remaining candidate compounds would provide additional benefit to a low dose of glucocorticoid, we stimulated THP-1 cells with TNF and treated them with serially diluted compounds and 100 nM DEX (Figure 4A–C). The addition of DEX significantly reduced the CXCL8 production at all of the effective doses of 1-1, but was only effective at the lower doses of 1–2. There was minimal benefit to the effect of 3–1. Next we formally addressed whether there was an additive or synergistic effect with these three compounds and DEX. The compounds and DEX were titrated at the same molarity and in culture with TNF stimulated THP-1 cells and the release of CXCL8 was measured (Figure 5A–C). Here the IC_50_ for 1-1 and dexamethasone for TNF stimulated CXCL8 release were 968 and 300 nM respectively (Figure 5A). The four CXCL8 levels below the maximum plateau were used to calculate an isobologram (Figure 5D). The relative potency values for compound 1-1 are near the origin, demonstrating synergy. The values for 1-2 were modestly synergistic, however the values for 3-1 were not all consistent with synergy. Hence compound 1-1 was considered for testing in primary human cells (Figure 5D).

### Compound 1-1 Suppresses Chemokine Production by RA FLS and Is Synergistic With GC

**FIGURE 6:**
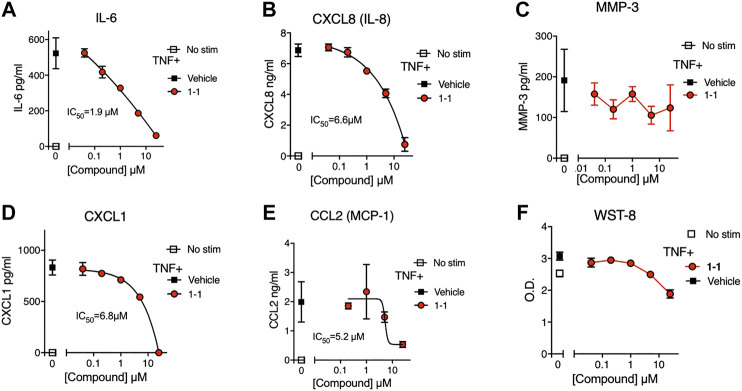
Compound 1-1 suppresses chemokine and cytokine release by TNF stimulated RA FLS; **(A–E)** Chemokine and cytokine production by RA FLS stimulated with 1 ng/ml of TNF for overnight with graded dilutions of compound 1–1. The supernatants were assayed for IL-6; **(A)**, CXCL8; **(B)**, MMP-3; **(C)**, CXCL1; **(D)** and CCL2; **(E)**. **(F)** Cell viability was assessed by WST-8 assay. DMSO was 0.1% of the final concentration as vehicle. Data are represented as mean ± SD. The IC_50_ values are shown. Data are representative of two independent experiments showing similar results.

**FIGURE 7:**
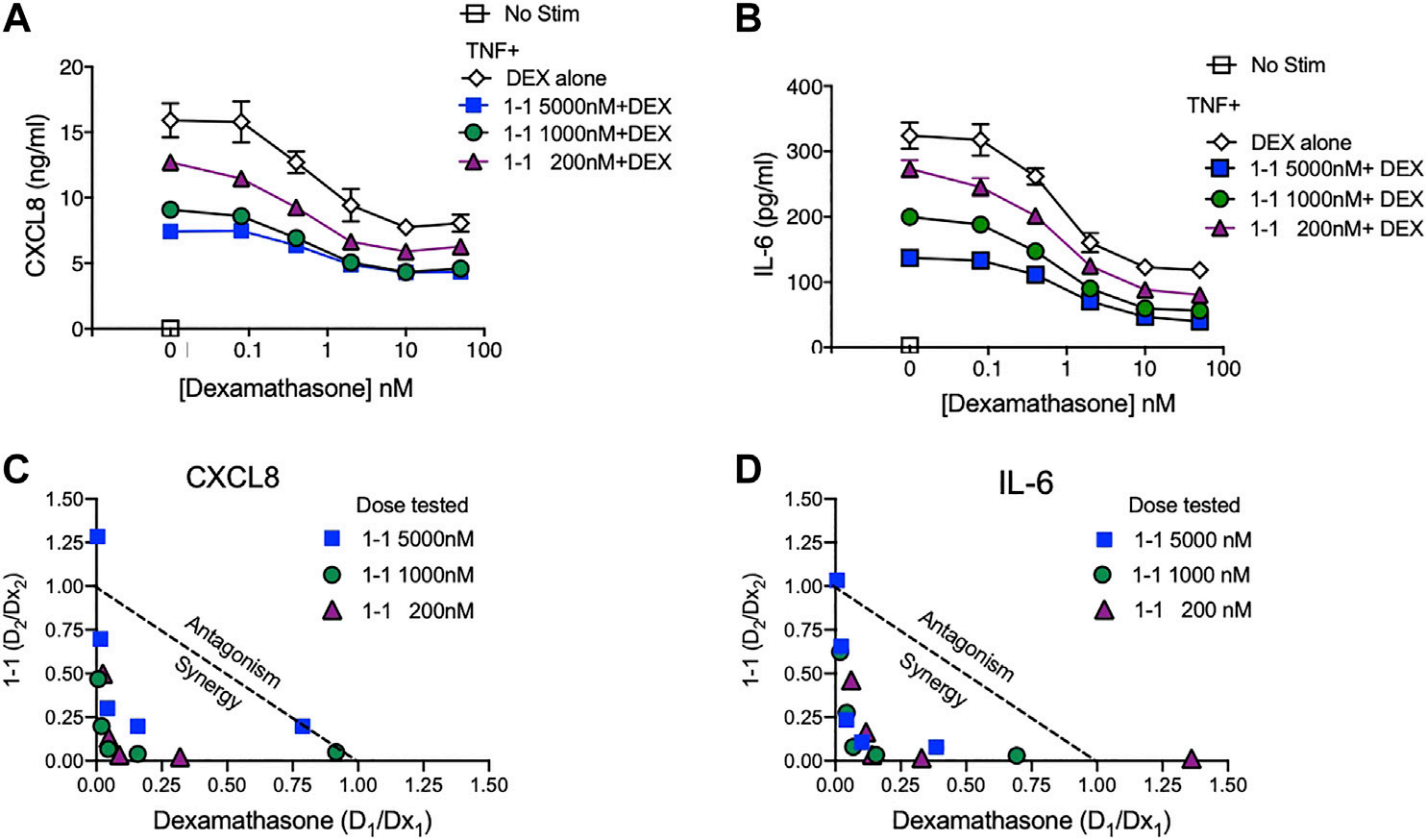
Synergistic suppression of CXCL8 and IL-6 production by compound 1–1 and DEX. Levels of CXCL8; **(A)** and IL-6; **(B)** secreted into the supernatant by RA FLS stimulated overnight with 1 ng/ml of TNF and treated with the indicated concentrations of 1–1 and DEX were measured by ELISA. Data are represented as mean ± SEM. Potency ratios were calculated and presented as isobolograms for CXCL8; **(C)** and IL-6; **(D)**. The dotted line represents additivity between DEX and the compounds. Data are representative of two independent experiments showing similar results.

In the pathogenesis of rheumatoid arthritis, fibroblast-like synoviocytes (RA FLS) are a primary source of inflammatory cytokines and chemokines in inflamed joints (Firestein and McInnes, 2017). We analyzed the immunomodulatory effects and cytotoxicity of compound 1-1 on RA FLS. Compound 1-1 dose-dependently suppressed CXCL1, CXCL8, CCL2, and IL-6 production induced by TNF, but not MMP-3 production (Figure 6A–E). Compound 1-1 also showed low cytotoxicity in RA FLS, similar to the THP-1 cells (Figure 6F). To assess the synergistic effect of compound 1-1 with DEX in RA FLS, we analyzed IL-6 and CXCL8 suppression by co-titrating DEX and compound 1-1 in cultures with TNF stimulated RA FLS (Figure 7A, B). Isobolograms of the potency ratios indicated that compound 1-1 also showed synergistic effects with DEX in RA FLS for both IL-6 and CXCL8 release (Figure 7C, D).

## Discussion

Recently there has been significant development of biologic and non-biologic disease modifying anti-rheumatic drugs (DMARDs), which have moved into clinical application (Kesharwani et al., 2019). However, GCs and NSAIDs remain indispensable as bridge therapy or co-therapy with DMARDs (Hua et al., 2020). Here, we identified compounds that reduced NF-κB activity and chemokine/cytokine secretion induced by potent inflammatory stimuli, and acted synergistically with GCs. We selected compounds based on a classic “Top X” approach for bioactivity, but also informed our selection of lead candidates by the frequency of hits in the larger chemotype clusters. We have previously improved our hit confirmation rates by using a similar chemoinformatic enrichment method for hit selection (Pu et al., 2012). These compounds were not toxic to the monocytic cell line or to primary human cells in culture.

In the past we used a broad cell based screening approach to identify compounds with a desired function and did not limit the potential targets. By re-analyzing the data from our two existing HTS assays, we could identify 1824 candidate compounds with activity similar to GCs from the >134,000 compounds that overlapped between the two HTS libraries (Chan et al., 2013; Chan et al., 2017). Once these 1824 compounds were selected, we reassessed their effects on the kinetics of NF-κB activity at two different time points, which we had identified as peak activity for the dose of LPS chosen and then later in the decay phase of the LPS stimulation. There were known NF-κB inhibitory compounds included in the library, including IKK inhibitors and polymyxin B, which suppressed NF-κB activity at both timepoints. However, the GC in the library suppressed NF-κB activity only at the later time point and not at the peak LPS stimulated NF-κB activity. Hence we chose 122 compounds using a Top X selection approach that suppressed NF-κB activity at one or both time points (Pu et al., 2012).

Cell-based phenotypic assays generally rely on multiple biological pathways to show the desired effect and can be prone to false positives. However, we increased our confidence in compound selection by adding a chemoinformatic approach and clustered the compounds by scaffold (chemotype) (Pu et al., 2012). The advantages of this approach had been demonstrated in our previous reports, that is, a large cluster suggests that there are replications in favorable biological activity of the candidate compounds, and the negative data afford a structure-activity relationship within the family to guide future strategic structure-activity designs. Interestingly, chemotype cluster #1 was the largest one in the chemical library. Although we have not yet defined the molecular target, which is a limitation of using a cell-based assay, we have some clues based on 11 hit compounds and more than a hundred compounds with a shared chemotype in the larger library that lacked suppressive activity for future structure-activity experimental design.

The lead compound 1–1 has been previously described to have bioactivity in another system. This compound was discovered as one of a chemotype cluster of pyrazolo [3,4 *d*]pyrimidines to be a positive allosteric modulator of the metabotropic glutamate receptor subtype 4 (mGluR4) (Niswender et al., 2008). Metabotropic glutamate (mGlu) receptors are a family of G protein-coupled receptors activated by the neurotransmitter glutamate. This activity, including modulation of Ca^++^ flux, was characterized in cellular experiments. Although direct binding to a target was not performed, others have indicated that a different mGLU4 positive allosteric modulator (PAM) could inhibit TNF release from LPS stimulated microglial cells in culture (Ponnazhagan et al., 2016). Other cell types such as dendritic cells may be affected by this class of drug (Volpi et al., 2016). A PAM of mGlu4 has been demonstrated to activate noncanonical mGluR4 signaling in dendritic cells (DC) and induce a tolerogenic functional phenotype through IDO1, an immunoregulator and reduce neuroinflammation in a murine model of multiple sclerosis (Volpi et al., 2016).

As many small molecules have multiple targets with different binding affinities and this may be the case with 1–1. We started with the premise that a compound that reduced NF-κB signaling may be beneficial to lower the dose of steroids needed to attain an anti-inflammatory effect. GCs bind the glucocorticoid receptor (GR) and form a GC-GR complex when they transition into the nucleus, and then regulate gene expression by transactivation (TA) with binding of GC-GR complex to gene promoters, and by transrepression (TR). Since most of the adverse effects induced by GCs were mediated by metabolic effects via TA by GC-GR complex, several groups tried to identify selective GR activators (SEGRA) from natural products by assessing their binding to GR and their activity in transactivation and transrepression assays (Schacke et al., 2004; Lesovaya et al., 2015). As we showed here, our lead compounds showed similar inhibitory kinetics with GCs, suggesting that an inhibitory mechanism might be shared with GCs. However, our lead compound did not inhibit all NF-κB associated activity as seen in the minimal inhibition of MMP-3 production by FLS. The promotor region for MMP-3 includes binding sites for the activator proteins (AP) -1, the polyomavirus enhancer-A binding protein-3 (PEA3), and other transcription factors that may continue to induce MMP-3 transcription despite partial NF-κB inhibition (Newby, 2006; Fanjul-Fernandez et al., 2010). Identifying the mechanism of action of our compounds and comparing with GCs would be the next step to further drug development.

To minimize the adverse events of GCs, decreasing the dosage of GCs with the concomitant use of other agents was examined to maintain the therapeutic efficacy. The lead compound 1-1 clearly demonstrated synergistic effects with DEX (Figures 5–7), suggesting that this compound may have a dose-sparing effect. These findings indicate the possibility of reducing the dose of GCs, but also potentially enhancing the effects of endogenous GCs secreted physiologically. Other agents have been reported to reduce inflammation in models of arthritis that are insufficient alone, but utilize a complementary pathway that favorably modulates the activity of a known therapeutic agent. For example, the receptor tyrosine phosphatase sigma (PTPRS) activating decoy protein attenuated severity of arthritis when combined with low dose of a TNF inhibitor (Svensson et al., 2020), but was insufficient in itself to have an effect. In addition, we have reported that the combination of an inhibitor of cell proliferation and a TNF inhibitor exerted synergistic effects without reducing immune responses (Hosoya et al., 2016).

In summary, we successfully identified novel anti-inflammatory compounds by an immune phenotype based screening. The lead compounds showed anti-inflammatory effects with minimal if any cellular cytotoxicity. By analyzing multiple potential inflammatory stimuli, including LPS, TNF and IL-1ß we narrowed the candidates to those that reduced chemokine secretion to all tested stimuli. The lead 1*H*-pyrazolo [3,4 *d*] pyrimidin-4-amine compound (1–1) had an IC_50_ at the micromolar level in RA FLS comparable to that in the human monocytic cell line THP-1. Furthermore synergistic anti-inflammatory effects with dexamethasone and compound 1-1 were demonstrated in both THP-1 cells and primary human RA FLS. Our study provided the foundation for future studies including specific mechanism of action studies, target identification, and additional preclinical assessments of FLS migration, invasion, proliferation and apoptosis should be performed using the lead compound. A synoviocyte-directed therapy as evaluated here with compound 1-1combined with a targeted biologic strategy, like an anti-TNF monoclonal antibody, could be a successful strategy with less toxicity than current therapeutic approaches (Feldmann and Maini, 2015).

## **Data Availability Statement**

The raw data supporting the conclusions of this article will be made available by the authors, without undue reservation.

## Ethics Statement

The studies involving human participants were reviewed and approved by institutional review board at Tokyo Medical and Dental University, Tokyo, Japan. The patients/participants provided their written informed consent to participate in this study.

## Author Contributions

TH, NS, HC, DC, and MC designed research, interpreted data and drafted the manuscript. YF, SY, FL, HB, SY, and TH conducted experiments. TH, TH, NS, and MC performed statistical analyses. All authors contributed to discussions, and had opportunity to review and revise the manuscript.

## Funding

This study was partially supported by the National Institute of Health/National Institute of Allergy and Infectious Diseases under an Adjuvant Discovery Contract HHSN272201400051C (DAC). This work was supported in part by the Japan Research Foundation for Clinical Pharmacology (TH).

## Conflict of Interest

The authors declare that the research was conducted in the absence of any commercial or financial relationships that could be construed as a potential conflict of interest.

## Footnotes

b The mean CXCL8 induced by LPS was 185.3 pg/ml and was normalized as 100%.

c The mean CXCL8 induced by LPS was 201.1 pg/ml and was normalized as 100%.
