# Supplementary material for: Identification of Compounds With Glucocorticoid Sparing Effects on Suppression of Chemokine and Cytokine Production by Rheumatoid Arthritis Fibroblast-Like Synoviocytes
Source: Front Pharmacol. 2020 Dec 17;11:607713. doi: 10.3389/fphar.2020.607713 (PMC7773657; doi:10.3389/fphar.2020.607713)
Supplement: Supplementary file 1 [file table1.pdf]

Supplemental Table 1. Commercially Purchased Hit Compounds

Chemo-type	Com-pound	MW (g/mol)	Vendor	Catalog ID	Structure
1	1-1	321	ChemDiv	K402-0050	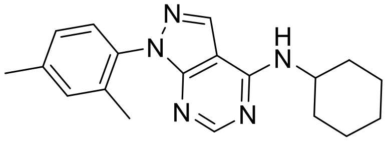
1	1-2	295	ChemDiv	K402-0009	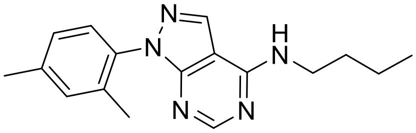
3	3-1	261	ChemBridge	5402902	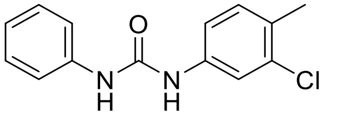
5	5-1	276	ChemBridge	5302354	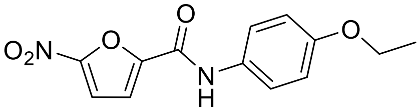
5	5-2	300	ChemBridge	5346896	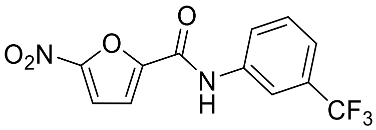
5	5-3	312	ChemBridge	5350540	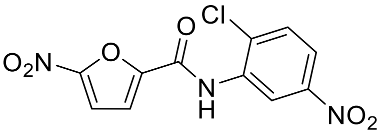
13	13-1	454	ChemDiv	5282-0180	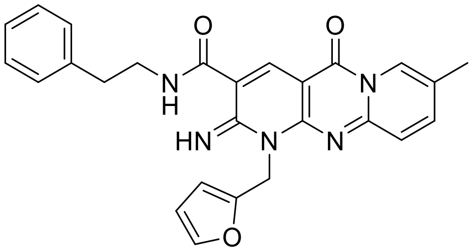
13	13-2	454	ChemDiv	5282-0176	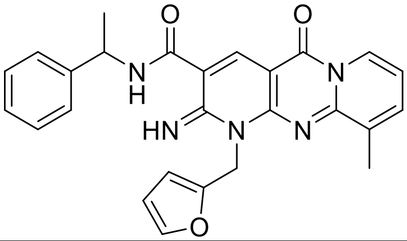
17	17-1	417	ChemDiv	G274-0173	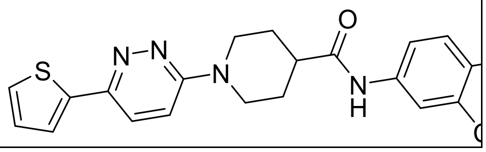
