# Supplementary figures and images for: Identification of Compounds With Glucocorticoid Sparing Effects on Suppression of Chemokine and Cytokine Production by Rheumatoid Arthritis Fibroblast-Like Synoviocytes
Source: Front Pharmacol. 2020 Dec 17;11:607713. doi: 10.3389/fphar.2020.607713 (PMC7773657; doi:10.3389/fphar.2020.607713)

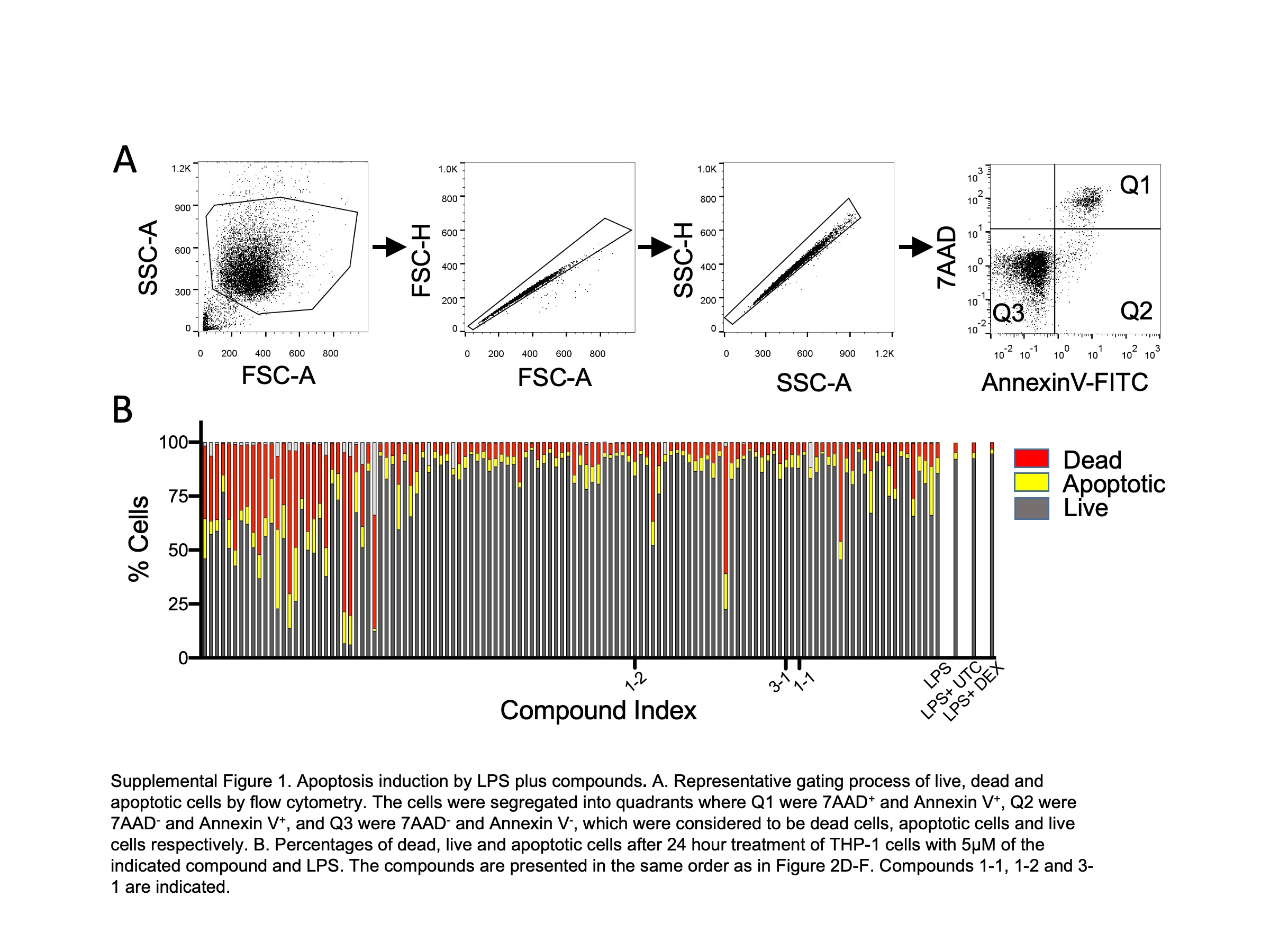

Supplement: Supplementary file 2 [file image1.jpeg]

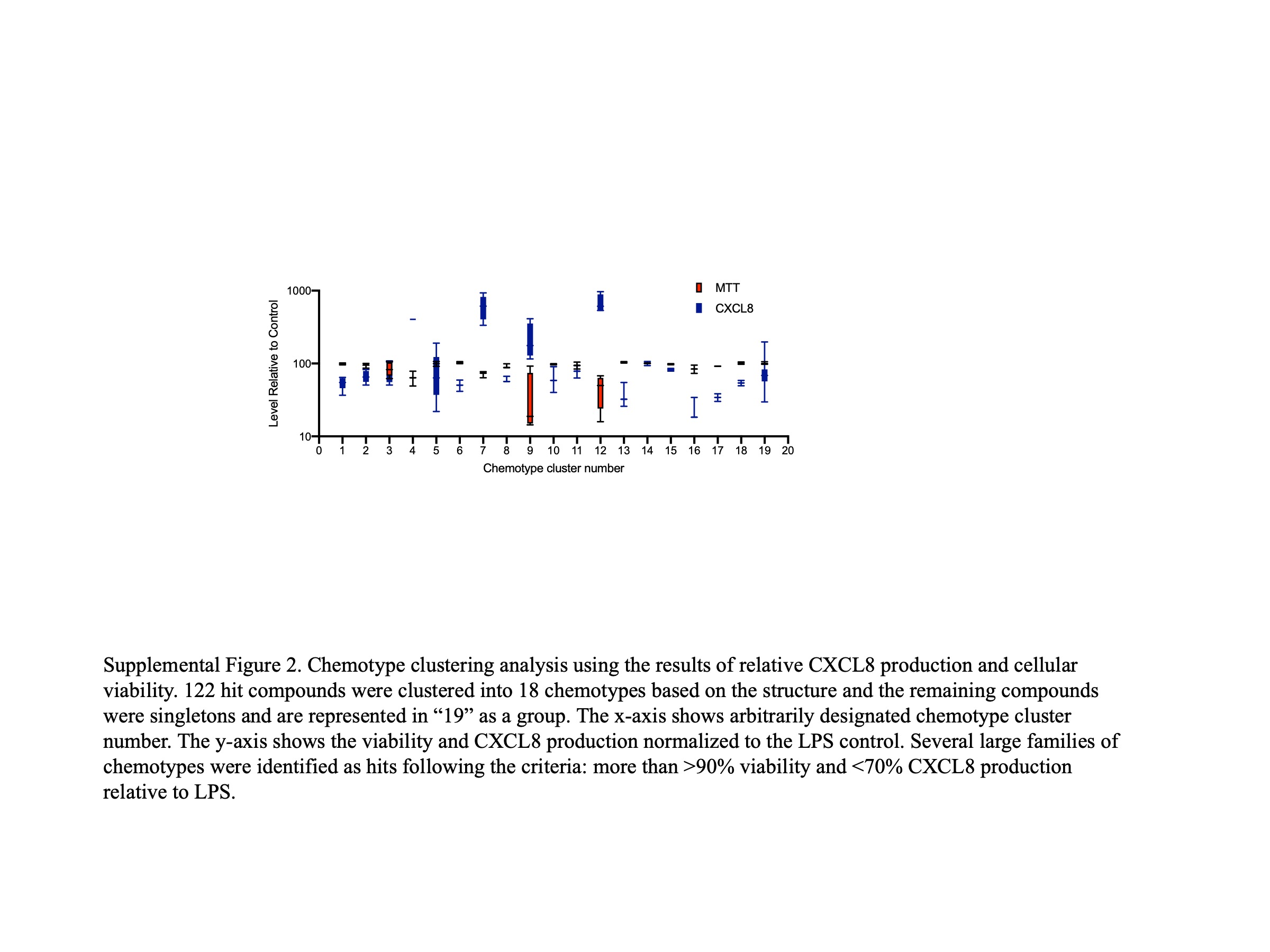

Supplement: Supplementary file 3 [file image2.jpeg]
